# *Lactobacillus casei* CCFM1074 Alleviates Collagen-Induced Arthritis in Rats *via* Balancing Treg/Th17 and Modulating the Metabolites and Gut Microbiota

**DOI:** 10.3389/fimmu.2021.680073

**Published:** 2021-05-17

**Authors:** Zhexin Fan, R. Paul Ross, Catherine Stanton, Bao Hou, Jianxin Zhao, Hao Zhang, Bo Yang, Wei Chen

**Affiliations:** ^1^ State Key Laboratory of Food Science and Technology, Jiangnan University, Wuxi, China; ^2^ School of Food Science and Technology, Jiangnan University, Wuxi, China; ^3^ International Joint Research Laboratory for Pharmabiotics & Antibiotic Resistance, Jiangnan University, Wuxi, China; ^4^ APC Microbiome Ireland, University College Cork, Cork, Ireland; ^5^ Teagasc Food Research Centre, Moorepark, Co., Cork, Ireland; ^6^ Department of Basic Medicine, Jiangnan University, Wuxi, China; ^7^ National Engineering Research Center for Functional Food, Jiangnan University, Wuxi, China; ^8^ Wuxi Translational Medicine Research Center and Jiangsu Translational Medicine Research Institute Wuxi Branch, Wuxi, China

**Keywords:** *Lactobacillus casei*, arthritis, immunity responses, gut microbiota, serum metabolome, genome

## Abstract

Gut microbiota and their influence on metabolites are receiving increasing attentions in autoimmune diseases including rheumatoid arthritis (RA). Probiotics become a promising manipulator to prevent or attenuate the progression of arthritis, some evidences suggesting that lactobacilli treatment influence the responses to RA therapy but the underlying mechanisms are limited. By using a collagen-induced arthritis (CIA) rats, the study assessed the effects of two *L. casei* strains (CCFM1074, CCFM1075) on the immune responses, gut microbiota and plasma metabolites *via* an integrated cross-omics approach including fecal 16S rRNA high-throughput sequencing and plasma metabolomics. The genome of the two strains was analyzed and compared using whole-genome sequencing approach to further confirm biology functions. CCFM1074 reduced arthritic symptoms while CCFM1075 did not, though both strains down-regulated the plasma IL-6 and Th17 cells proportion. CCFM1074 enhanced the proportion of Treg cells in mesenteric lymph nodes which was significantly associated with SCFAs upregulation, as well as with genomic evidence that CCFM1074 possesses more functional genes involved in carbohydrate metabolism. Moreover, CCFM1074 regulated the gut microbiota, including modulating community structure, decreasing the abundance of *Alistipes* and *Parabacteroides* and increasing the abundance of *Oscillibacter*. The differential metabolites modulated by CCFM1074 including eicosapentaenoic acid and docosapentaenoic acid which involved in unsaturated fatty acids metabolism. Furthermore, alterations of gut microbial community were correlated with the plasma metabolome. In summary, *L. casei* CCFM1074 alleviated arthritis *via* rebalancing gut microbiota, immune responses and plasma metabolites.

## Introduction

Rheumatoid arthritis (RA) is a chronic autoimmune-mediated inflammatory disorder characterized by synovitis and joint destruction. In addition to genetic and environmental factors responsible for the development of RA, there is clear and strong evidence for an involvement of gut microbiota in RA progression. This is supported by facts that dysbiosis of gut microbiota preceded RA symptoms, particularly *Prevotella copri* strongly correlated with disease, and the perturbation was normalized after methotrexate treatment (1–3). Recent numerous studies found significant alteration in gut microbiota in RA patients, which correlated with serologic features in clinical (4). Remarkably, transplantation of fecal microbiota from RA patients aggravates arthritis in collagen-induced arthritis (CIA) mice, further demonstrating that dysbiosis in gut microbiota are not only a result of the disease, but also a reason for the progression of RA (5). Investigations on gut microbiota suggest probiotics might be an alternative therapeutic target to prevent or attenuate arthritis.

More recently, several animal and clinical studies supported the benefits of probiotics in alleviating arthritis. Of note, oral administration of *L. casei* ATCC 334, a type strain for *L. casei*, significantly suppressed the induction of arthritis, restored the gut microbiota and reduced oxidative stress (6–8). And another commercial strain, *L. casei* 01, exhibited considerable capacity to alleviate inflammation, which was associated with suppressing Th1 immune response and the oral tolerance in mice model of arthritis (9). Additionally, it has been reported that some non-commercial *L. casei* strains also exerted suppressive effects on arthritis, for instance, the mixture of CCFM1074 and CCFM1075 was reported in our previous study (10, 11). Regardless of the usage and dosage of *L. casei* strains and the heterogeneity across the studies, the previous findings highlighted the similar immunoregulative properties of various *L. casei* strains. Given probiotics are generally accepted to host- and strain-specific, it is therefore important to have an in-depth understanding of the specified crosstalk between probiotics, gut microbiota and mucosa immune in the host.

The present study aimed to compare the effects of *L. casei* in relieving arthritis. In hence, two strains of *L. casei* (CCFM1074, CCFM1075) on immune responses, gut microbiota and plasma metabolites were compared using a CIA rats. Furthermore, genomic comparison among the two strains and ATCC334 was performed to gain better insight of the functional characteristics, which may provide a novel theory for rapidly screening of anti-arthritis candidate probiotics.

## Materials and Methods

### Animals

Six-week-old female Wistar rats (purchased from Beijing Vitalstar Biotechnology Co., Ltd) were housed in a specific pathogen-free conditions at 23 ± 2°C, 50% to 60% humidity, and at a 12/12 h light/dark rhythm. Rats received water and standard chow ad libitum across the trial. After the animals were anesthetized with isoflurane, the blood was taken from the heart, and then the animals were killed by breaking their necks. Animal experimentation was approved by the Ethics Committee of the Jiangnan University [JN.No20190315W1040527(17)].

### *L. casei* Strains and Treatment Protocols

CCFM1074 and CCFM1075, isolated from human feces, were deposited at Culture Collection of Food Microbiology (CCFM) at Jiangnan University. The two strains were cultured in DeMan-Rogosa-Sharpe (MRS) medium at 37°C. The culture was centrifuged at 5000 g for 10 min; the cell pellets were washed twice with saline and re-suspended at 8.5 × 10^9^ CFU/mL in 30% (w/w) sucrose solution, and stored at –80°C prior to use; the bacterial suspensions were re-centrifuged (5000 g, 10 min) and re-suspended in saline to get oral preparation before daily oral treatment. After a one-week habituation period, rats were randomly divided into five groups: control (vehicle-treated with saline 1.5 mL/day) group, CIA (vehicle-treated with saline 1.5 mL/day) group, methotrexate (MTX)-treated (6 mg/kg/week) group (6), CCFM1074 (treated with CCFM1074, 8.5 × 10^9^ CFU/day) group and CCFM1075 (treated with CCFM1075, 8.5 × 10^9^ CFU/day) group, eight rats per group. In addition, the rats in MTX group were fed with 1.5 mL saline per day before induction of arthritis.

### Induction and Assessment of Collagen-Induced Arthritis

CIA was induced and assessed following the method described previously (12). Briefly, bovine type II collagen solution (20 mg/mL, Chondrex, Redmon, WA, USA) were emulsified with incomplete Freund’s adjuvant (0.05M, Chondrex, Redmon, WA, USA) at a ratio of 1:1, and 150 μl emulsion were subcutaneously injected into the rat tail base at day 1. After seven days, rats received a booster immunization with 150 μl emulsion at tail. The control rats were subcutaneously injected with same volume of sterile saline. Arthritic symptoms developed at paw and joint, the paws thickness was measured using calipers and the severity of symptoms was assessed by a quantitative clinical score as following: 0, no redness and swelling signs; 1, mild erythema and swelling limited to individual digits, or mild swelling of ankle or wrist; 2, moderate erythema and swelling of ankle or wrist; 3, severe erythema and swelling of the entire paw; 4, maximally erythema and swelling of limb.

### Histological Assessment

The joints were fixed in 10% formalin for 48 h and decalcified in 10% EDTA-2Na, embedded in paraffin, and sliced into 5-μm-thick sections. The sections were staining with hematoxylin and eosin (H&E) to assess the histologic changes. Slides were qualitatively evaluated using the following parameters: synovial hyperplasia (pannus formation), cell exudate, depletion of cartilage and bone erosion.

### Micro-Computed Tomography (Micro-CT) Scanning

The entire hind limb was scanned using Quantum GX2 micro-CT scanner (PerkinElmer, Shelton, Connecticut, USA) to capture anatomical images with high resolution to assess bone health. The projections were obtained using a high resolution scan mode that 144 μm voxel size, 90 kV tube voltage, 88 μA current and 4 min scan time. Analyze software (AnalyzeDirect, Overland Park, Kansas, USA) was used to reconstruct the 3-dimensional (3D) structure of joints, and the same anatomic location of ankle joint were cropped to verification of bone destruction and the joint damage be visualized.

### Quantified Cytokines

After sacrificing the rats, blood was collected and centrifugated (3000g, 15 min) for plasma collection. Interleukin-10 (IL-10), Interleukin-6 (IL-6), Interleukin-1β (IL-1β), and tumor necrosis factor-α (TNF-α) in plasma were determined using commercial ELISA kits (R&D Systems, MN, USA) according to the manufacturer’s protocols.

### Flow Cytometry Analysis

Mesenteric lymph nodes (MLN) were dissected and collected. For preparation of a single-cell suspension, MLN were mechanically mashed and filtered through a 100-μm nylon strainer in PBS containing 0.1% NaN_3_. Cell staining was performed as previously described (13). Briefly, cells were incubated with FITC-conjugated anti-rats CD4 monoclonal antibody (mAb) and APC-conjugated anti-rats CD25 mAb for surface staining at 4°C for 30 min. For Tregs detection, the cells were fixed and permeabilized at room temperature for 1 h, and then stained with PE-conjugated anti-rats/mouse Foxp3 mAb in Foxp3/Transcription staining buffer. For Th17 cell detection, MLN single-cell resuspended in complete RPMI1640 medium with cell activation cocktail for 5 h in a CO_2_ incubator. Stimulated cells were washed twice with staining buffer and performed surface staining protocol, and then carried out intracellular cytokine staining with PE-conjugated anti-rats IL17A mAb for 1 h at room temperature. PE-conjugated rat IgG2a was used as the isotype control for Foxp3 and IL-17A staining. All those conjugated antibodies and reagents were purchased from eBioscience (San Diego, CA, USA). Cells were acquired using Attune NxT Acoustic Focusing Cytometer (Model AFC2 Thermo, CA, USA) and the data were analyzed using FlowJo software (Tree Star, Ashland, OR, USA).

### 16S rRNA V3-V4 High-Throughput Sequencing and Bioinformatic Analyses

The DNA were extracted from 500-mg fecal samples using the FastDNA Spin Kit (MP Biomedical, Irvine, CA, USA) in accordance with manufacturer’s protocol. Each DNA sample was amplified for V3-V4 regions of the bacterial 16S rRNA using 341/806 primer pairs. The PCR amplification was carried out according to protocol descried previously (14). The amplified products were purified through 1% agarose gel using Gel Extraction kit (Tiangen, Beijing, China). The purified samples were quantified using the Qubit 2.0 (Life Technologies, CA, USA) and amended to equimolar solution, pooled and library preparation, followed by sequenced in Illumina MiSeq platform as per the manufacturer’s instructions.

Sequenced raw data were demultiplexed and filtered using QIIME2 pipeline with DADA2 (15, 16). The representative sequences were picked to taxonomic categories against to the Silva database with a 97% threshold. PICRUSt2 were applied to infer the functional characterizers of the gut microbiota (17). Alpha diversity was evaluated by Simpson and Shannon indexes, evenness and Faith’s phylogenetic diversity (Faith_PD). Beta diversity was assessed by weighted UniFrac distances and visualized with principal coordinate analysis (PCoA), and the difference were assessed by PERMANOVA test. Linear discriminant analysis (LDA) Effect Size (LEfSe) analysis was performed to mine the discriminatory taxa among groups (LDA > 3.0, p < 0.05). Spearman’s correlation analyzes were performed to evaluate the potentially relevant associations on gut microbiota with responses in plasma. The correlation-network was plotted using Cytoscape (|r| > 0.3).

### Quantified Short-Chain Fatty Acids in Feces

Fecal samples were vacuum freeze-dried and short-chain fatty acids (SCFAs) were extracted using the method described by Wang et al. (18). Briefly speaking, after digesting the sample, the supernatant is collected after centrifugation. After the water is removed, the gas phase vial is added to the machine for analysis. The SCFAs were analyzed using a GC-MS system and the chromatographic conditions were set up as previous method (18).

### Preparation of Plasma Samples for Untargeted Metabolomics Analysis

Plasma were stored at –80°C and thawed at 4°C before analysis. Three times volume of methanol were added to plasma samples to precipitate protein, vortexed for 30 s, ultrasonicated at 4°C for 10 min, placed at –20°C for 1 h, and centrifugated for 15 min (15,000*g*, 4°C). The supernatant (80 μl) was transferred to a new Eppendorf tube and dried in a centrifugal vacuum evaporator. The samples were reconstituted dissolved in 80% methanol and analyzed in a random way. Quality control (QC) samples were prepared by pooling an equal volume of reconstitution from every plasma sample and were analyzed after every 10 plasma samples. QC samples can monitor the system stability and reproducibility. Blank sample (80% methanol) was used for background subtraction and noise removal.

## Liquid Chromatography (LC) and Mass Spectrometry (MS)

The metabolites in plasma were analyzed using a ItiMateU-3000 UPLC system (Thermo Fisher Scientific, MA, USA) coupled with a high-resolution Q-Exactive mass spectrometer in full scan mode (Thermo Fisher Scientific, MA, USA). A 2.1 × 100 mm reverse-phase Waters Acquity UPLC T3 column (Waters, MA, USA) was used for chromatographic separation at 35°C. The mobile phase consisted of 0.1% aquenous formic acid (A) and 0.5 mM ammonium acetate-acetonitrile (B) with a gradient elution: 0–1.0 min (5%, B); 1.0–10 min (5–99%, B); 10–12 min (99%, B) and 12–15 min (5%, B). The flowrate was 0.3 mL min^−1^ and the injection volume was 2 μl. The MS system with a heated electrospray ionization (ESI) source was operated in the positive and negative ion modes with a full-scan range covered 151 to 2000 m/z and 70 to 1050 m/z, respectively. And the parameters were set as follows: 1.50 kV source voltage and 250°C capillary temperature. Capillary voltages for negative and positive ionization modes were set at 2500 and 3500 V, respectively.

### Data Processing

MS data were processed by Compound Discovery 3.1 (Thermo Fisher Scientific, MA, USA) using untargeted metabolic workflow with default parameters, which included data extraction, background features filtered out, peak identification, deconvolution, alignment and integration, and metabolite identification assignment *via* mzCloud, HMDB, KEGG, and ChemSpider databases. The data processing filtrated out metabolic features that appeared < 50% of the QC samples and that >30% RSD of the QC samples, and the mass tolerance was 5 ppm. Metabolic features that whose m/z ratios could not match the masses in the above databases were filtrated out. The processed data including peak area, retention time (RT), molecular weight (MW), and identified or unknown compound were integrated in an excel. Multivariate analysis was performed in SIMCA software (Umetrics, Umea, Sweden). After normalized peak area with the method of unit vector scaling, the unsupervised principal component analysis (PCA) were performed to investigate the overview of metabolome variation among groups and drew by the ggbiplot package of R software, and supervised orthogonal partial least-squares discriminant analysis (OPLS-DA) models were introduced to probe the features that responsible for classifying groups (19). Permutation test with 200 were used to check for overfitting of the models. Metabolic features with variable important in projection (VIP, VIP>1 and p < 0.05) were considered as potential differential biomarkers. Metabolic pathway analysis was performed in MetaboAnalyst 5.0 (https://www.metaboanalyst.ca/) with rattus norvegicus pathway library.

### Bacterial Genome Sequencing, Assembly and Annotation

The genomic DNA of each strain was extracted and purified using a bacterial DNA extraction kit (OMEGA, Biotech, Doraville, GA, USA). The complete genomes of CCFM1074 and CCFM1075 were sequenced in Illumina Hiseq × 10 platform by Shanghai Majorbio Bio-pharm Technology Co., Ltd (Shanghai, China). Genomic sequence of *L. casei* ATCC334 was achieved from NCBI. The genome assembly and annotation were performed as previously reported (20). In briefly, genome assembly was carried out using SOAPdenovo v3.04 with default options and the corresponding functional genes were annotated by blast against cluster of KEGG database using Diamond (e-value ≤ 1e -5).

### Statistical Analysis

Comparison between community composition and alpha-diversity was determined by Kruskal-Wallis non-parametric test followed by Dunn’s multiple comparisons using GraphPad Prism 8 (Graphpad Software Inc., San Diego, CA, USA). The nonparametric multivariate analysis of variance was performed to assess the differences in beta-diversity of gut community among groups using Vegan package (R, version 3.6.5). Mann-Whitney U test was performed between two groups in Metabolomic analysis. Visualization of the correlation network in Cytoscape (version 3.6.0). The absolute value of Spearman correlation coefficient > 0.3 were plotted. Two-tailed p < 0.05 were considered significant. Data are expressed as mean ± SEM or median with interquartile range, with p value less than 0.05 considered statically significant.

## Results

### *L. casei* Treatments Alleviated the Development of CIA

**Figure 1:**
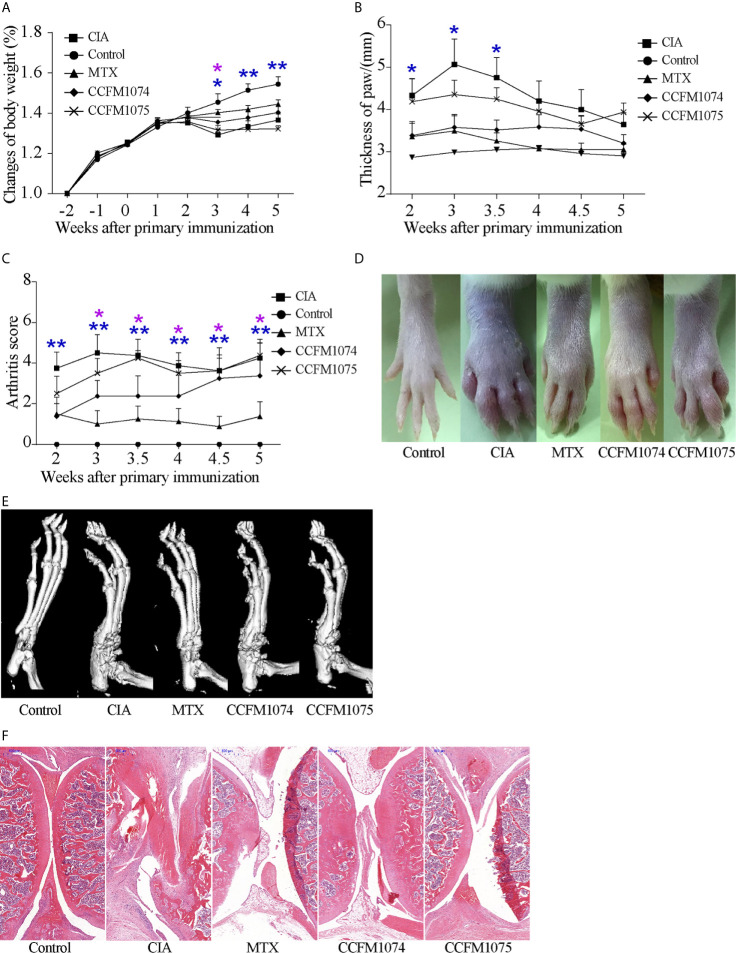
Effects of *L. casei* on CIA in rats. **(A)** Change of body weight, **(B)** Thickness of the paws, **(C)** arthritis score. Representative photographs of paw **(D)** and representative microCT images of paw **(E)**. control (vehicle-treated with saline 1.5 mL/day) group, CIA (vehicle-treated with saline 1.5 mL/day) group, methotrexate (MTX)-treated (6 mg/kg/week) group, CCFM1074 (treated with CCFM1074, 8.5 × 10^9^ CFU/day) group and CCFM1075 (treated with CCFM1075, 8.5 × 10^9^ CFU/day) group, eight rats per group. The representative histopathological sections of knee were determined by H&E staining (scale bar 500 μm) **(F)**. *p<0.05, **p<0.01 (two-way ANOVA with Dunnett’s multiple comparisons test). Blue *, CIA *vs*. Control; purple *, CIA *vs*. MTX.

Responding to immunization with bovine type II collagen, rats exhibited arthritis-associated symptoms such as erythema, swollen in paw and joints and weight loss (**Figures 1A–D**). The changes of body weight prior to paw swelling and the inflammatory clinical feature maintained during the whole progress. After 8-week intervention, the rats orally administered with CCFM1074 or MTX revealed less aggravated redness and swollen in paw, while rats in CCFM1075 group showed an indistinctive phenotype comprised with those in model group, particularly in the changes of body weight.

The results of micro-CT showed that both CCFM1074 and CCFM1075 reduced the bone erosion in ankles, the micro-CT image showed an intact joint architecture and smooth bone surface in CCFM1074 and CCFM1075 treated rats, whereas the joint in CIA rats presents with compromised joint integrity (**Figure 1E**). H&E staining analysis further validated the suppressive effects of *L. casei*. Compared with CIA group, the knee joint of rats in CCFM1074 and CCFM1075 groups exhibited significantly pathological improvements regarding to less inflammatory cells infiltrate in synovial tissue and less destruction in cartilage in *L. casei* treated groups, as well as in rats treated with MTX group (**Figure 1F**).

### *L. casei* Treatments Regulated Systemic and Local Immune Response

**Figure 2:**
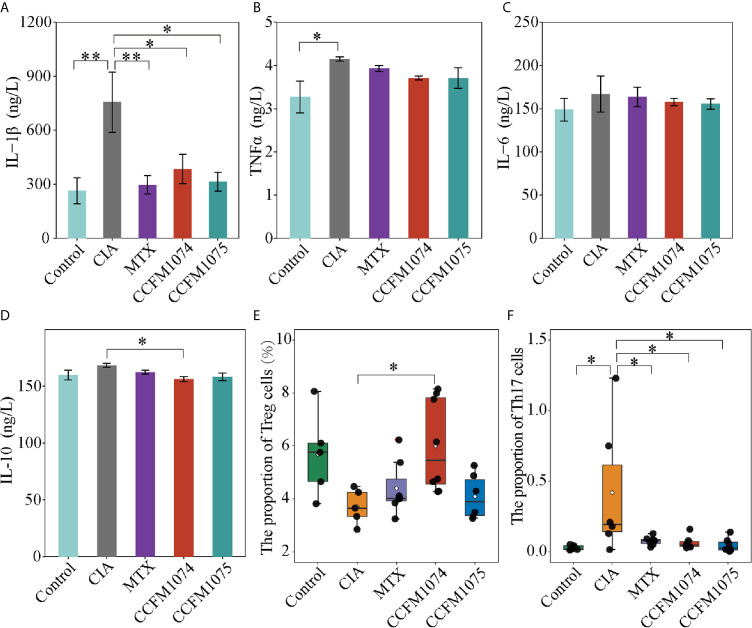
Effects of *L. casei* on inflammatory responses in rats. The levels of IL-1β **(A)**, TNFα **(B)**, IL-6 **(C)** and IL-10 **(D)** in plasma were determined by ELISA. The percentage of Treg cells **(E)** and Th17 cells **(F)** in MLN were determined by flow cytometry. control (vehicle-treated with saline 1.5 mL/day) group, CIA (vehicle-treated with saline 1.5 mL/day) group, methotrexate (MTX)-treated (6 mg/kg/week) group, CCFM1074 (treated with CCFM1074, 8.5 × 10^9^ CFU/day) group and CCFM1075 (treated with CCFM1075, 8.5 × 10^9^ CFU/day) group, eight rats per group. *p<0.05, **p<0.01 (one-way ANOVA test with Tukey’s multiple comparisons test).

Cytokines in plasma were measured to assess the systemic immune response to inflammation, and pro-inflammatory cytokines (IL-6, TNFα, and IL-1β) play a key inductive role, whereas IL-10 is a resistive anti-inflammatory cytokine. CIA rats showed a significant higher level of IL-1β and TNFα (**Figures 2A, B** **)**, while IL-6 and IL-10 only exhibited a mild increase compared with control rats (**Figures 2C, D** **)**. Oral treatment with CCFM1074 and CCFM1075 significantly inhibited the production of IL-1β, but not significance in reducing IL-6 and TNFα. In addition, CCFM1074 effectively impeded the increase of IL-10 induced by articular inflammation and the suppressive effects of the two *L. casei* strains on systemic inflammation were comparable to drug MTX.

Immune responses in extra-intestinal were partly driven by proliferation and differentiation of immunocyte in intra-intestinal. To investigate the functional effects of *L. casei* on local immune cells in intestinal niche, the T cells subpopulation in MLN were analyzed by flow cytometry. Treg frequency in CD4+ T cells tended to decrease and yet the fraction of Th17 cells was significantly increased in CIA rats compared with rats in control group (**Figures 2E, F** **)**. CCFM1074 treatment significantly enhanced the expansion of Treg cells in MLN, but the increase of Treg was imperceptible in CCFM1075 treated rats. Th17 cells in MLN from the rats treated with CCFM1074 and CCFM1075 significantly recovered to the lower interval which was similar to that in control rats. In addition, similar but weaker effects were observed for alteration of subpopulation of CD4+ T cells in MTX treated rats compared with rats in CCFM1074 group.

### *L. casei* Affected Gut Microbiota With Strain-Dependence

**Figure 3:**
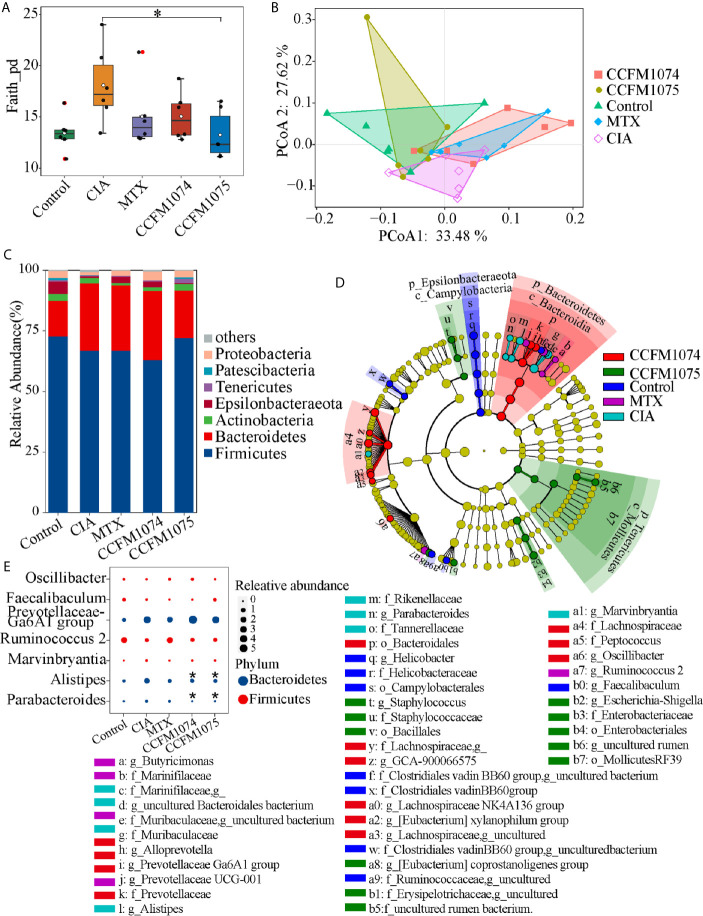
Effects of *L. casei* on gut microbiota in CIA rats. The effects of *L. casei* on alpha diversity of Faith_pd **(A)** and PCoA of beta diversity **(B)** after *L. casei* treatment. The gut microbial compositions profiles at the phylum level **(C)**. The differential microbial taxa among groups were shown as cladogram by LEfSe analyze **(D)**. Changes of the relative abundance at the genus level **(E)**. control (vehicle-treated with saline 1.5 mL/day) group, CIA (vehicle-treated with saline 1.5 mL/day) group, methotrexate (MTX)-treated (6 mg/kg/week) group, CCFM1074 (treated with CCFM1074, 8.5 × 10^9^ CFU/day) group and CCFM1075 (treated with CCFM1075, 8.5 × 10^9^ CFU/day) group, eight rats per group. *p < 0.05 (Kruskal-Wallis test with Dunn’s multiple comparisons test). *, vs. CIA.

Gut dysbiosis is a risk factor for arthritis, this study attempted to clarify whether the perturbed gut microbes could be recovered by *L. casei* administration. The sequence counts ranged from 12174 to 55463. Differences of gut microbial taxon within-samples were analyzed by alpha diversity and phylogenetic diversity. Species richness (observed species) and alpha-diversity (Shannon and Evenness indexes) showed no significant differences between the model and control groups (**Supplementary Figures 1A–C**), and the phylogenetic diversity (Faith’s phylogenetic diversity) was 1.34-fold increase in model group (p = 0.073) (**Figure 3A**). Compared with model group, the phylogenetic diversity was 83.25% (p > 0.05) and 73.24% (p = 0.024) lower in CCFM1074 and CCFM1075 groups, respectively. PCoA suggested that the microbial composition in model group was significantly distinguished from others (**Figure 3B**). Moreover, the distribution of samples from rats treated with MTX overlapped with that from CCFM1074 group (p = 0.447), and the cluster for CCFM1075 was tend to that of control group. In addition, there was a significant difference in cluster for CCFM1074 and CCFM1075 (p = 0.027). This evidence suggested that the microbial structure changed after arthritis development, and CCFM1075 partly normalized the microbial community.

At the phylum level, Bacteroidetes significantly increased in both CIA and CCFM1074 treated rats compared to control rats, which only showed a negligible increase in CCFM1075 group (**Figure 3C**). Other main phylum taxa (Actinobacteria, Firmicutes, and Tenericutes) showed a recovery after oral administration with CCFM1075. In addition, compared to MTX, CCFM1075 significantly altered the relative abundance of Epsilonbacteraeota and Tenericutes. 26 genera were identified as specific differences by LEfSe analysis among groups, and among which six genera were uncultured bacteria (**Figure 3D**). Both CCFM1074 and CCFM1075 treatments significantly decreased the relative abundance of *Alistipes* and *Parabacteroides*, while neither of them normalized the abundance of Prevotellaceae Ga6A1 group, Ruminococcus 2 and Marvinbryantia (**Figure 3E**).

**Figure 4:**
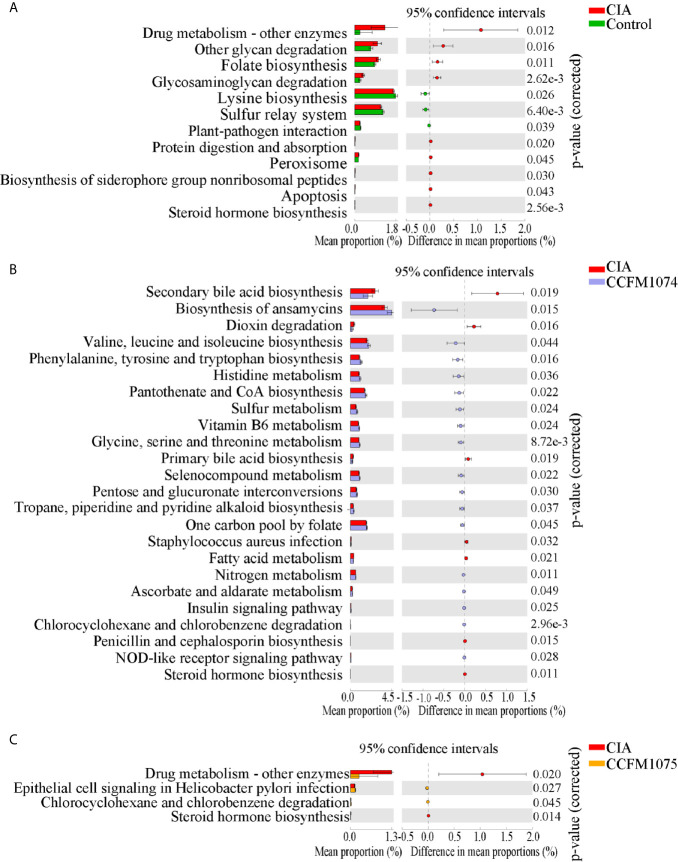
Effects of *L. casei* on predicted metabolic pathways in CIA rats. The predicted metabolic pathways in Control **(A)**, CCFM1074 **(B)** and CCFM1075 **(C)** compared with CIA. control (vehicle-treated with saline 1.5 mL/day) group, CIA (vehicle-treated with saline 1.5 mL/day) group, methotrexate (MTX)-treated (6 mg/kg/week) group, CCFM1074 (treated with CCFM1074, 8.5 × 10^9^ CFU/day) group and CCFM1075 (treated with CCFM1075, 8.5 × 10^9^ CFU/day) group, eight rats per group. Significant differences were tested using Welch’s t test.

PICRUSt2 were used to further explore the functional pathways associated with gut taxa. Twelve functional pathways were significantly altered when the rats were challenged with arthritis (**Figure 4A**). The functions of drug metabolism, other glycan degradation, folate biosynthesis, glycosaminoglycan degradation, protein digestion and absorption, peroxisome, biosynthesis of siderophore group nonribosomal peptides, apoptosis and steroid hormone biosynthesis were enriched in CIA group, while lysine biosynthesis and sulfur relay system were decreased. Twenty-four and four referred pathways changed after CCFM1074 and CCFM1075 treatments, respectively. (**Figures 4B, C** **)**. And some pathways such as secondary bile acid biosynthesis, histidine metabolism and vitamin B6 metabolism were resumed at both CCFM1074 and MTX groups (**Supplementary Figure 1D**). Specifically, steroid hormone biosynthesis pathway was significantly diminished in the two *L. casei* groups.

### *L. casei* Treatments Modulated the SCFAs in Fecal Samples

**Figure 5:**
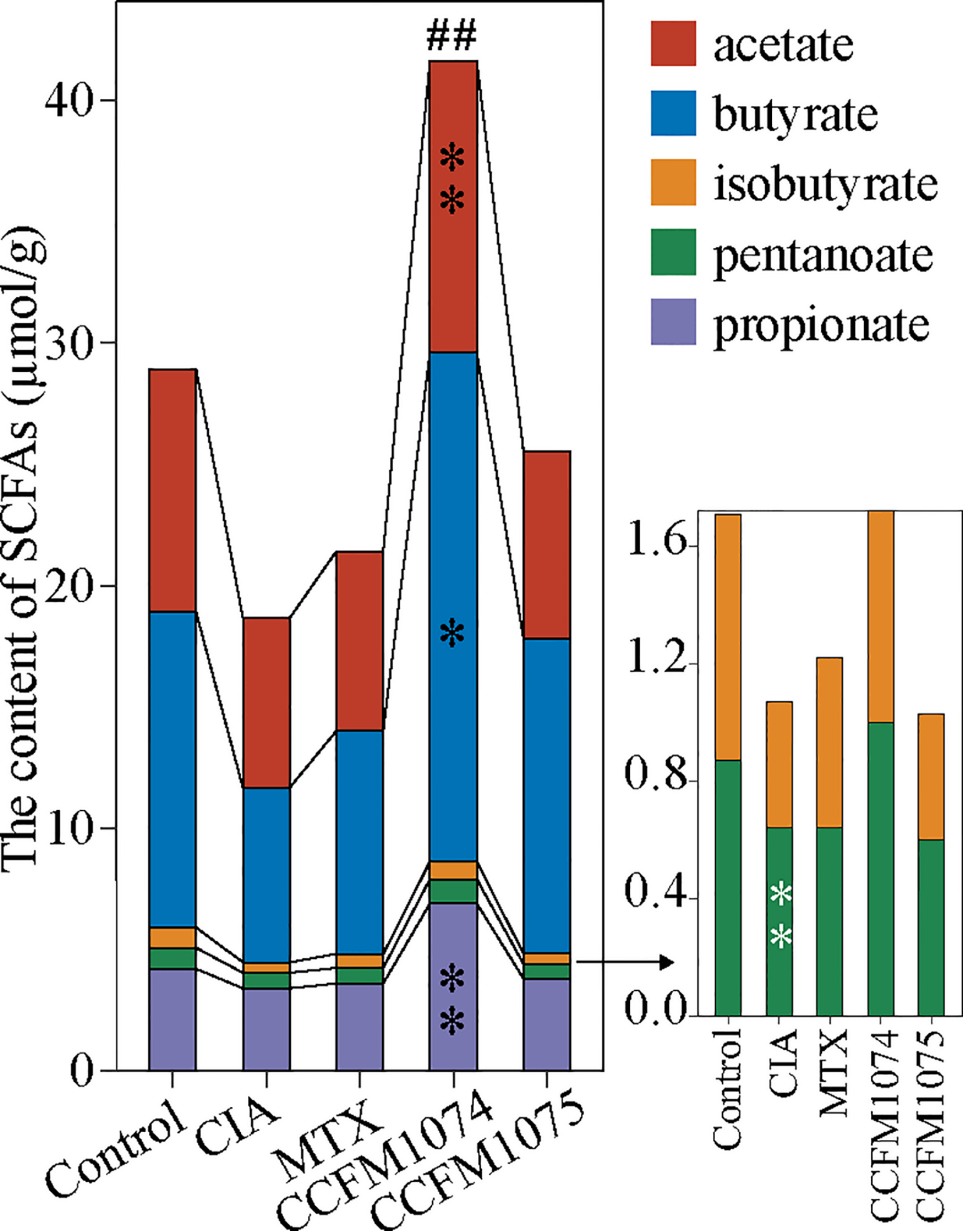
Effects of *L. casei* on SCFAs production in CIA rats. control (vehicle-treated with saline 1.5 mL/day) group, CIA (vehicle-treated with saline 1.5 mL/day) group, methotrexate (MTX)-treated (6 mg/kg/week) group, CCFM1074 (treated with CCFM1074, 8.5 × 10^9^ CFU/day) group and CCFM1075 (treated with CCFM1075, 8.5 × 10^9^ CFU/day) group, eight rats per group. *p<0.05, **p<0.01, ^##^ *p*<0.01 (one-way ANOVA test with Dunnett’s multiple comparisons test). Black *, *vs*. CIA; white *, *vs*. Control; ^##^, *vs*. CIA (total SCFAs).

SCFAs, especially butyrate and propionate, were well studied in the alleviating arthritis in murine models. It was expected that CIA rats had lower concentration of SCFAs compared to control rats, among which iso-butyrate was reduced significantly (**Figure 5**). The alterations of SCFAs were strain-dependent. CCFM1074 could significantly recovered total fecal SCFAs to normal levels, in contrast, CCFM1075 had no remarkable effects on SCFAs production although butyrate was 1.78 fold than that in CIA rats.

### *L. casei* Treatments Modulated the Plasma Metabolism

**Figure 6:**
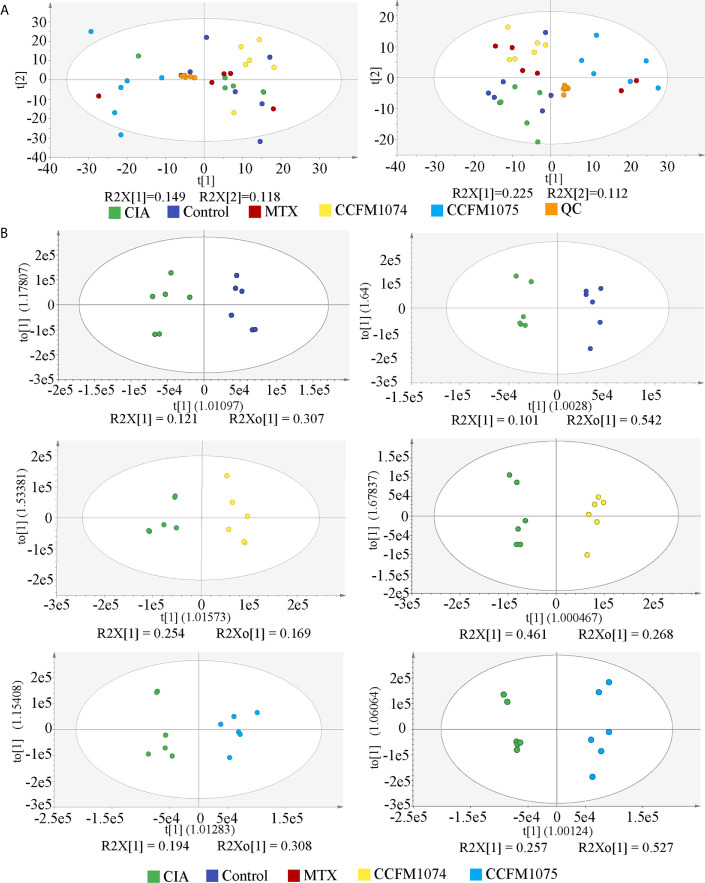
The PCA and OPLS-DA score plots of plasma samples among groups. **(A)** PCA score plots in ESI+ model (left panel) or ESI- model (right panel). **(B)** OPLS-DA score plots between two groups in ESI+ model (left panel) and ESI- model (right panel). control (vehicle-treated with saline 1.5 mL/day) group, CIA (vehicle-treated with saline 1.5 mL/day) group, methotrexate (MTX)-treated (6 mg/kg/week) group, CCFM1074 (treated with CCFM1074, 8.5 × 10^9^ CFU/day) group and CCFM1075 (treated with CCFM1075, 8.5 × 10^9^ CFU/day) group, eight rats per group.

Untargeted metabolomic with two ionization modes (ESI^+^ and ESI^-^) were used to analyze the metabolomic profiles of plasma. PCA of all the detected features showed a close clustering of QC samples indicating stability and repeatability of the instrument system, as well as a visual separation between CIA and *L. casei* groups in either ESI^+^ or ESI^-^ model, suggesting that administration of *L. casei* remarkably influenced the metabolic features in plasma (**Figure 6A**). In total, 1270 features were yielded in ESI^+^ and 611 features were obtained in ESI^-^. OPLS-DA were applied to discriminate the differential features, and the results showed a clear segregation between the control rats and CIA rats, and between *L. casei* treated rats and CIA rats (**Figure 6B**), as well as between MTX treated rats and CIA rats (**Supplementary Figure 2A**). The 200-test permutation test further validated good predictabilities of OPLS-DA (**Supplementary Figure 2B**). Based on accurate masses, MS/MS fragments and matched from self-built commercial databases mzCloud, there were 41, 17, and 51 features identified as differential biomarkers (VIP > 1 and *p* < 0.05) in MTX, CCFM1074, and CCFM1075 groups, respectively, compared with model rats (**Supplementary Table S1**).

**Figure 7:**
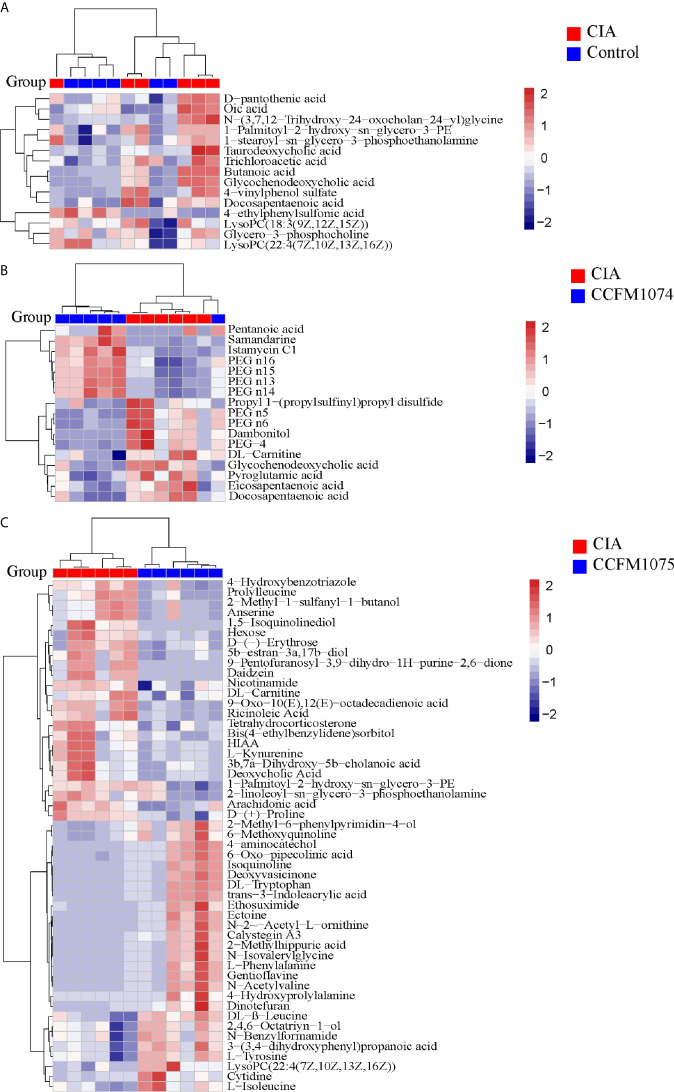
Analyzes of differential metabolites in plasma between groups. Heat map of metabolites between CIA and Control **(A)**, between CIA and CCFM1074 **(B)**, between CIA and CCFM1075 **(C)**. control (vehicle-treated with saline 1.5 mL/day) group, CIA (vehicle-treated with saline 1.5 mL/day) group, methotrexate (MTX)-treated (6 mg/kg/week) group, CCFM1074 (treated with CCFM1074, 8.5 × 10^9^ CFU/day) group and CCFM1075 (treated with CCFM1075, 8.5 × 10^9^ CFU/day) group, eight rats per group. The differential metabolites were identified as VIP >1 and *p*<0.05.

**Figure 8:**
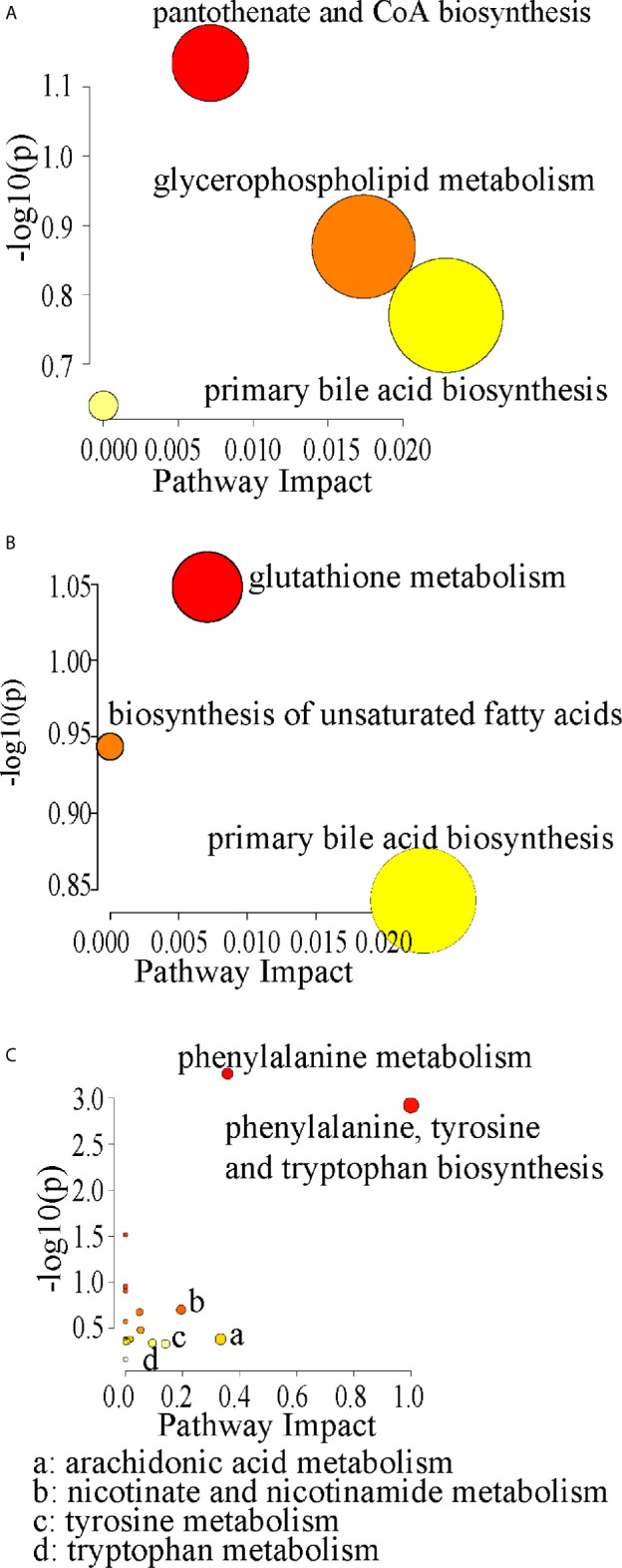
Pathway analyzes of the differential metabolites in CIA **(A)**, in CCFM1074 **(B)** and in CCFM1075 **(C)**. control (vehicle-treated with saline 1.5 mL/day) group, CIA (vehicle-treated with saline 1.5 mL/day) group, methotrexate (MTX)-treated (6 mg/kg/week) group, CCFM1074 (treated with CCFM1074, 8.5 × 10^9^ CFU/day) group and CCFM1075 (treated with CCFM1075, 8.5 × 10^9^ CFU/day) group, eight rats per group.

Compared with control rats, the differential metabolites D-pantothenic acid, glycochenodeoxycholic acid, taurodeoxycholic acid, LysoPC(18:3(9Z,12Z,15Z)), and LysoPC(22:4(7Z,10Z,13Z,16Z)) were involved in pantothenate and CoA biosynthesis, glycerophospholipid metabolism and primary bile acid biosynthesis in model rats (pathway impact < 0.1) (**Figures 7A** and **8A**). After administration with CCFM1074, the altered metabolites (pyroglutamic acid, eicosapentaenoic acid, glycochenodeoxycholic acid) were involved in glutathione metabolism, biosynthesis of unsaturated fatty acids, primary bile acid biosynthesis (pathway impact < 0.1) (**Figures 7B** and **8B**). Differently, the altered important metabolic pathways of CCFM1075 group were phenylalanine, tyrosine and tryptophan biosynthesis, phenylalanine metabolism, arachidonic acid metabolism, nicotinate and nicotinamide metabolism, tyrosine metabolism and tryptophan metabolism (**Figures 7C** and **8C**).

### Correlation of Microbial Community, Metabolites and Immune Responses

**Figure 9:**
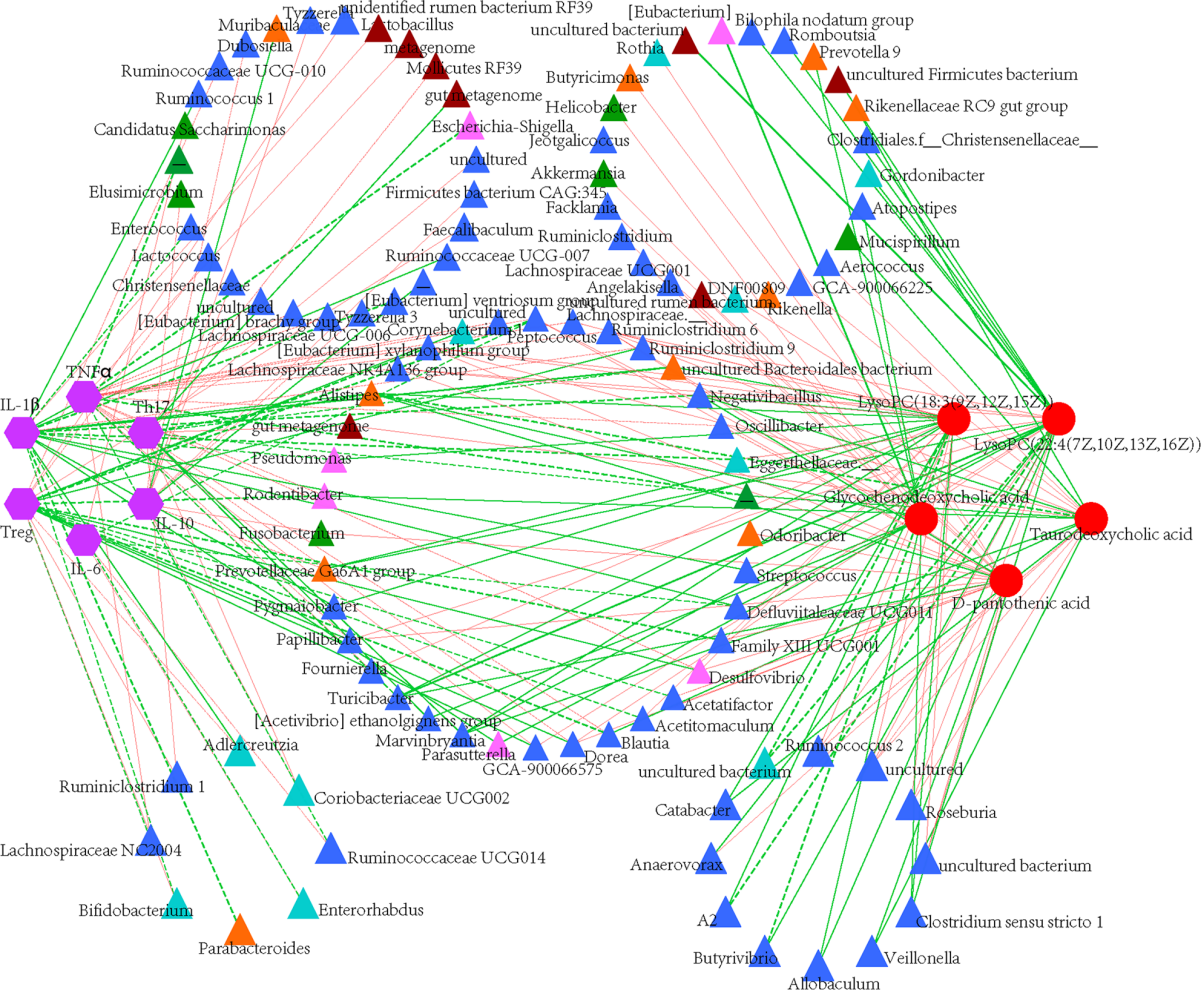
Construction of correlation-network diagram. Correlation analyze of gut microbial genus (relative abundance > 0.1%), cytokines and identified differential metabolites that involved in pathway analyzes. The edge colors indicate positive (red) or negative (green) correlations. Thickness of edge represents correlations and the color indicate positive (green) and negative (red) correlations, and dotted line indicated significant correlations.

Spearman’s correlation analyzes were used to further assess the potentially relevant correlation on immunological reactions, microbial taxa and plasma metabolites. 44 genera were significantly correlated with immunological indexes, of which *Rodentibacter*, *[Eubacterium] xylanophilum* group, *Parabacteroides* and *Blautia* showed inverse correlation in immune activated or immunosuppressive responses (**Figure 9**). Specifically, *Peptococcus* and *Defluviitaleaceae* UCG-011 were the most significantly genera correlated with immunological responses. Compared with immunological reactions, more gut taxa were correlated with plasma metabolites although only eight bacterial genera correlated significantly with metabolites, suggesting the correlation between microbial community and immunological reactions was stronger and more significant than that between microbiota and plasma metabolites, while the later was more complex. Furthermore, D-pantothenic acid mainly negatively correlated with gut microbiota.

### Genomic Functional Differences and Similarities Among *L. casei*

In the present study, CCFM1074 potentially alleviated the arthritis. *L. casei* ATCC334 was reported to significantly suppress the induction and progression of arthritis in rats (6). It is reasonably speculated that *L. casei* strains with the ability to suppress arthritis may have some similar functional genes. Though genes coding carbohydrate metabolism were the most different categories on KEGG annotation, the number of associated genes in CCFM1074, CCFM1075 and ATCC334 were 215, 191 and 210, respectively. In addition, the main subsystems of carbohydrate metabolism were listed in **Table 1**. Furthermore, **Table 2** lists the 14 genes shared by CCFM1074 and ATCC334 which absenting in CCFM1075.

**Table 1:** Genes number of carbohydrate metabolism subsystems.

Subsystems Description	CCFM1074	CCFM1075	ATCC344
Galactose metabolism	39	28	33
Starch and sucrose metabolism	48	39	47
Pentose phosphate pathway	28	24	27
Fructose and mannose metabolism	40	34	33
Glycolysis/gluconeogenesis	41	36	43
Amino sugar and nucleotide sugar metabolism	44	36	38
Pyruvate metabolism	34	36	35

**Table 2:** The description of main subsystems in carbohydrate metabolism.

Gene name	Gene Description/ encoded endonuclease	KO Description
gatB	PTS system, galactitol-specific IIB component/ [EC:2.7.1.200]	Galactose metabolism
gatB	PTS system, galactitol-specific IIB component/ [EC:2.7.1.200]	Galactose metabolism
gatB	PTS system, galactitol-specific IIB component/ [EC:2.7.1.200]	Galactose metabolism
gatC	PTS system, galactitol-specific IIC component/-	Galactose metabolism
gatC	PTS system, galactitol-specific IIC component/-	Galactose metabolism
gatC	PTS system, galactitol-specific IIC component/-	Galactose metabolism
ebgA	evolved beta-galactosidase subunit alpha/ [EC:3.2.1.23]	Galactose metabolism
scrA	PTS system, sucrose-specific IIB component/ [EC:2.7.1.211]	Starch and sucrose metabolism
scrA	PTS system, sucrose-specific IIB component/ [EC:2.7.1.211]	Starch and sucrose metabolism
crr	PTS system, sugar-specific IIA component/ [EC:2.7.1.-]	Starch and sucrose metabolism
crr	PTS system, sugar-specific IIA component/ [EC:2.7.1.-]	Starch and sucrose metabolism
talA	transaldolase/ [EC:2.2.1.2]	Pentose phosphate pathway
eda	2-dehydro-3-deoxyphosphogluconate aldolase/(4S)-4-hydroxy-2-oxoglutarate aldolase/ [EC:4.1.2.14 4.1.3.42]	Pentose phosphate pathway
adh	alcohol dehydrogenase/ [EC:1.1.1.1]	Glycolysis/Gluconeogenesis

## Discussion

In recent years, insights into the pathogenesis in RA has advanced considerably, supporting the substantial improvements with the biologic and small molecules drugs in clinical therapies. In fact, it is non-negligible that no or suboptimal therapeutic effects appeared in a considerable part of patients (21). Current research suggests that targeting the gut microbiota might be a strategy to optimize the therapeutic efficacy in arthritis, and previous studies found some lactobacilli were effective in treating RA. Therefore, understanding of the regulatory mechanism of Lactobacillus in RA is urgent to optimize the pharmacotherapy in clinical. Multiple lactobacilli species have been proposed for inhibiting RA development, including *L. casei* (6), *L. reuteri*, *L. rhamnosus* (22), and *L. acidophilus* (13), but the details of the species- and strain-based discrimination have not been studied. Our previous results showed that a *L. casei* cocktail alleviated arthritis through suppressing Th1 associated immune responses and modulating gut microbiota. Herein, multi-omics approaches were applied to assess the effects of the individual *L. casei* strain on modulating local and system immune responses, gut microbiota, SCFAs and plasma metabolites, as well as annotated the genes functions of *L. casei* attempt to clear the genomic characteristic of anti-arthritis lactobacilli.

To data, a great advances in mechanisms of *L. casei* in alleviating arthritis have been proven, including down-regulating Th1 effector functions, inhibiting proinflammatory cytokines generations, modulating gut microbiota and exerting antioxidant properties (7, 8, 23, 24). However, it is still a challenge to understand the shared or strain-specific characteristic of different *L. casei* strains against arthritis. Our current study showed that CCFM1074 and CCFM1075 exerted a non-distinctive and weak effects on systemic cytokines in plasma, with shared characteristics in inhibiting IL-1β and Th17 cells. IL-6 and TNFα are considered as key mediators in activating and recruiting macrophages, osteoclasts, chondrocytes and fibroblasts, and inducing the release of IL-1β, which directly mediates the synovial hypertrophy and cartilage damage during the development and maintenance of RA (25). IL-10, an anti-inflammatory effector cytokine, showed an augmented concentration in model rats, which was consistent with the clinical results that the concentration of IL-10 increased in both the plasma and synovia of RA patients (26). These results suggested that both CCFM1074 and CCFM1075 can inhibit the downstream cytokines of the cascaded inflammatory activities. Th1 cells and Th17 cells regarded as arthritogenic and autoreactive immunocytes which predominately promoted the inflammatory responses, while the Foxp3-expressing Treg cells exhibited an essential suppressive role on the development of arthritis (27). Specifically, the activation of Treg cells was much stronger in CCFM1074 compared with that in CCFM1075, supporting the positive effects in mitigating arthritic clinical symptoms of CCFM1074. Therefore, *L. casei* primarily influenced the local immunity in gut rather than plasma system immunity, which was consistent with the finding by *Gopalakrishnan* et al. that probiotic therapy can alter immunity locally (28). Commensals themselves and their metabolites profoundly influence the localized immune responses within the gut mucosa, and allow T cells skew to an altered T subsets (Th1/Th2 cells and Treg/Th17 cells). The SCFAs produced by commensals can shape local and systemic immunity responses, which, among diverse biological activities, have been shown to induce the differentiation and proliferation of Treg cells (29). The significant expansion of Treg cells in CCFM1074 group might be induced largely by the significant higher SCFAs, particularly butyrate. The SCFAs are produced by diverse microbes such as *Clostridium butyricum*, Lacnospiraceae, and Ruminococcaceae rather than *L. casei* per se. Furthermore, CCFM1074 and ATCC334 had more genes involved in carbohydrate metabolism, and made the strains easier to degrade saccharides and fibers which can provide the substrates for SCFAs-producing bacteria in the gut. The cross-feeding actions of ATCC334 also avail SCFAs increased *in vivo* and *in vitro* (30, 31). These results suggested abundant carbohydrate metabolism genes may be a genomic characteristic to screen Treg-promoted *L. casei* strains to alleviate arthritis.

The knowledge of distinct gut microbiota in RA patients from healthy individuals is an accepted truth in clinical. The bacterial community structure and composition altered significantly among groups although the two *L. casei* strains showed feeble effects on alpha diversity. A vast number of taxa changed differently after the two strains intervened, among which *Lactobacillus* showed a reduced trend in both CCFM1074 and CCFM1075 groups. The diminished *Lactobacillus* occurred in *L. casei* administrating groups may be due to their poor persistent colonization in gut. Similar results were reported by *Pan* et al., *L. casei* species was not enriched after oral administration of *L. casei* ATCC334, but other lactobacilli species were upregulated or downregulated. In addition, Lactobacillus expended in early RA patients. Therefore, the results suggested that Lactobacillus sensitive to the disease activities though it is difficult to clear whether the alteration was a cause or a result of arthritis. In general, gut microbiota structure in rats from CCFM1074 group trended to MTX-treated rats while CCFM1075 made it similar to control group. In addition to the alterations in community structure, there were similar changes in metabolome. Several altered pathways were overlapped between CCFM1074 and MTX groups, for instance, amino acid metabolism pathways (histidine metabolism and phenylalanine, tyrosine and tryptophan biosynthesis) and lipid metabolism (fatty acid metabolism and secondary bile acid biosynthesis), pantothenate and CoA biosynthesis and vitamin B6 metabolism, biosynthesis of ansamycins. Notedly, steroid hormone biosynthesis was changed in CCFM1074 group, as well as in CCFM1075 group.

Understanding the role of gut microbiota on the pathogenesis and development in RA is in its infancy. Several mechanisms on microbiota or its metabolites exerting nutritionally beneficial or toxic effects on immunity system have been reported. For instance, *Porphyromonas gingivalis* aggravated arthritis through activating Th17 immune responses and encoding peptidyl arginine deiminase that facilitated the generation of RA-related autoantigens citrullinated peptides (32, 33). Untargeted metabolomic approach, a strength and promising technology to identify and quantify the metabolites produced during cell and organisms’ activities, was applied to analyze the plasma metabolic profiles among groups. The concentration of docosapentaenoic acid (DPA) and eicosapentaenoic acid (EPA), typical long chain polyunsaturated fatty acid (PUFA), were increased in model group and normalized after CCFM1074 treatment. Previously literature reported that the increased omega-3 PUFA associated with the decreased risk of RA, and DPA in plasma might be a potentially screening marker for RA (34–36). DPA and EPA was considered as a new player to resolve inflammation although its limited concentration in various tissues. The explanation for the unexpected increase was that some mediators derived from omega-3 PUFA can promote and/or accelerate the inflammation resolution during acute inflammatory response (37). Plasma metabolites might be influenced by the inflammatory physiological status per se and the altered gut microbiota community. Therefore, the mediating mechanisms of gut microbiota in system metabolites was the key to manipulate the functions of probiotics in various gut environments, and health conditions among individuals.

In summary, CCFM1074 ameliorated arthritis by down-regulating the pro-inflammatory cytokines, rebalancing the ratio of Treg/Th17 cells, modulating gut microbiota and plasma metabolites (especially unsaturated fatty acids eicosapentaenoic acid and docosapentaenoic acid). CCFM1075 could not mitigating arthritis though down-regulating pro-inflammatory responses and normalizing gut microbiota, but without promoting anti-inflammatory Treg cells and mediating focused metabolites in plasma. Comprehensive data provided by multi-omics approach revealed that the alterations of gut microbiota intervened by *L. casei* were insufficient to prevent from arthritic inflammation, Treg cells in MLN and metabolites in plasma were pivotal to anti-arthritis of *L. casei*. The current study provided in-depth insights into the probiotics strategy in treating RA.

## Data Availability Statement

The data sets presented in this study can be found in online repositories. The names of the repository/repositories and accession number(s) can be found below: https://www.ncbi.nlm.nih.gov/, PRJNA705468 https://www.ncbi.nlm.nih.gov/, SRR12560096 https://www.ncbi.nlm.nih.gov/, SRR13908966.

## Ethics Statement

The animal study was reviewed and approved by the ethics committee of the Jiangnan University.

## Author Contributions

ZF: conceptualization, investigation, methodology, formal analysis, writing—original draft, and visualization. BY: conceptualization, methodology, validation, writing—review and editing, project administration, and funding acquisition. RR: writing—review and editing. CS: writing—review and editing. BH: methodology. JZ: validation, resources, data curation, and funding acquisition. HZ: conceptualization, methodology, validation, and funding acquisition. WC: resources, supervision, and funding acquisition. All authors contributed to the article and approved the submitted version.

## Funding

This research was supported by the National Natural Science Foundation of China (Nos. 32021005, 31820103010), National First-Class Discipline Program of Food Science and Technology (JUFSTR20180102), the Fundamental Research Funds for the Central Universities (JUSRP52003B), 111 Project (BP0719028), and Collaborative Innovation Center of Food Safety and Quality Control in Jiangsu Province.

## Conflict of Interest

CS was employed by Moorepark, Co.

The remaining authors declare that the research was conducted in the absence of any commercial or financial relationships that could be construed as a potential conflict of interest.

## Abbreviations

RA, rheumatoid arthritis; CIA, collagen-induced arthritis; MTX, methotrexate; H&E, hematoxylin and eosin; Micro-CT, micro-computed tomography; IL-10, interleukin-10; interleukin-6, IL-6; IL-1β, interleukin-1β; TNF-α, tumor necrosis factor-α; mAb, monoclonal antibody; Faith_PD, Faith’s phylogenetic diversity; LEfSe, linear discriminant analysis effect size; SCFAs, quantified short-chain fatty acids; QC, quality control; LC, liquid chromatography; MS, mass spectrometry; PCA, principal component analysis.
