# Supplementary figures and images for: Lactobacillus casei CCFM1074 Alleviates Collagen-Induced Arthritis in Rats via Balancing Treg/Th17 and Modulating the Metabolites and Gut Microbiota
Source: Front Immunol. 2021 May 17;12:680073. doi: 10.3389/fimmu.2021.680073 (PMC8165437; doi:10.3389/fimmu.2021.680073)

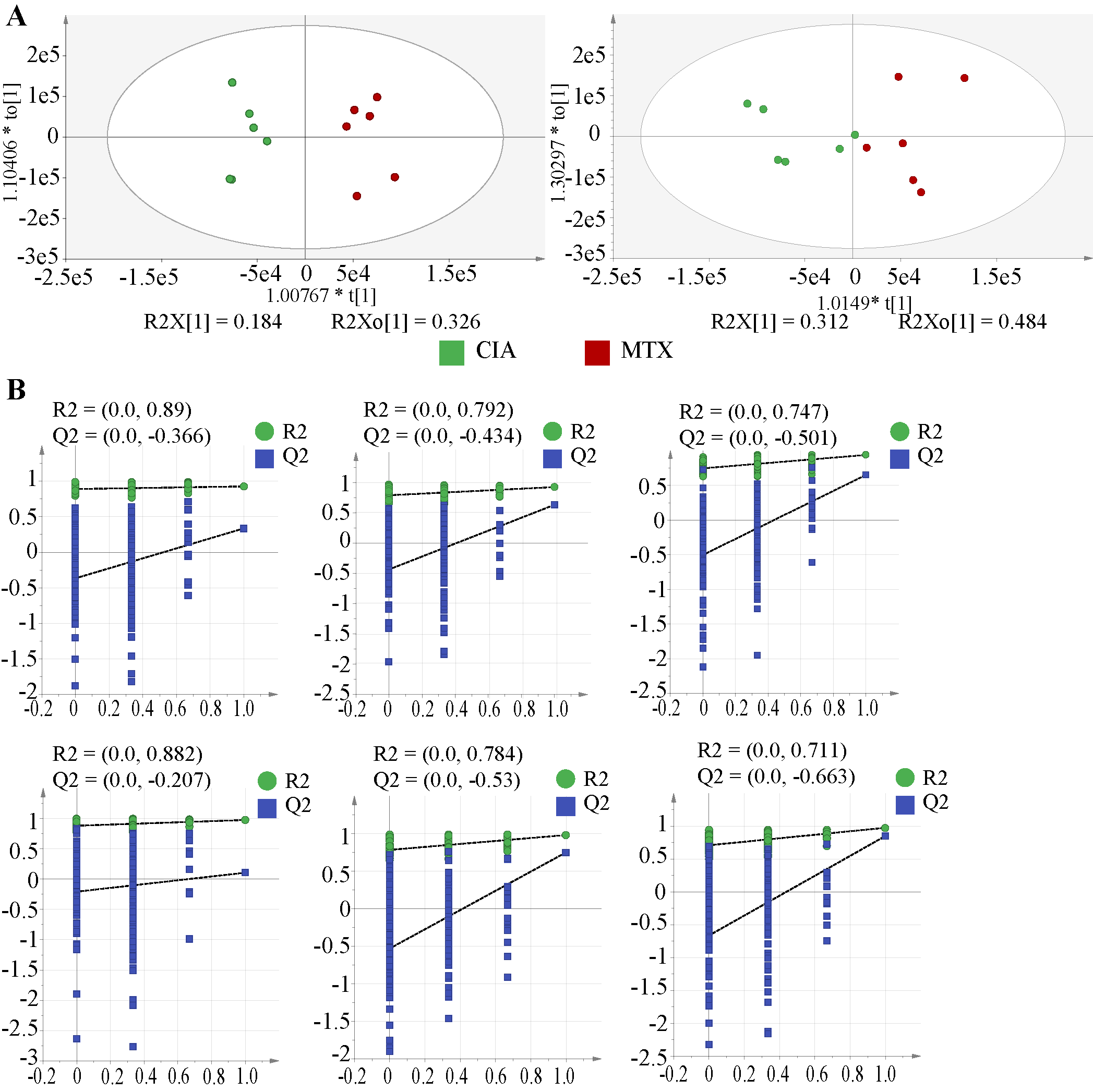

Supplement: Supplementary file 1 [file Image_1.tif]

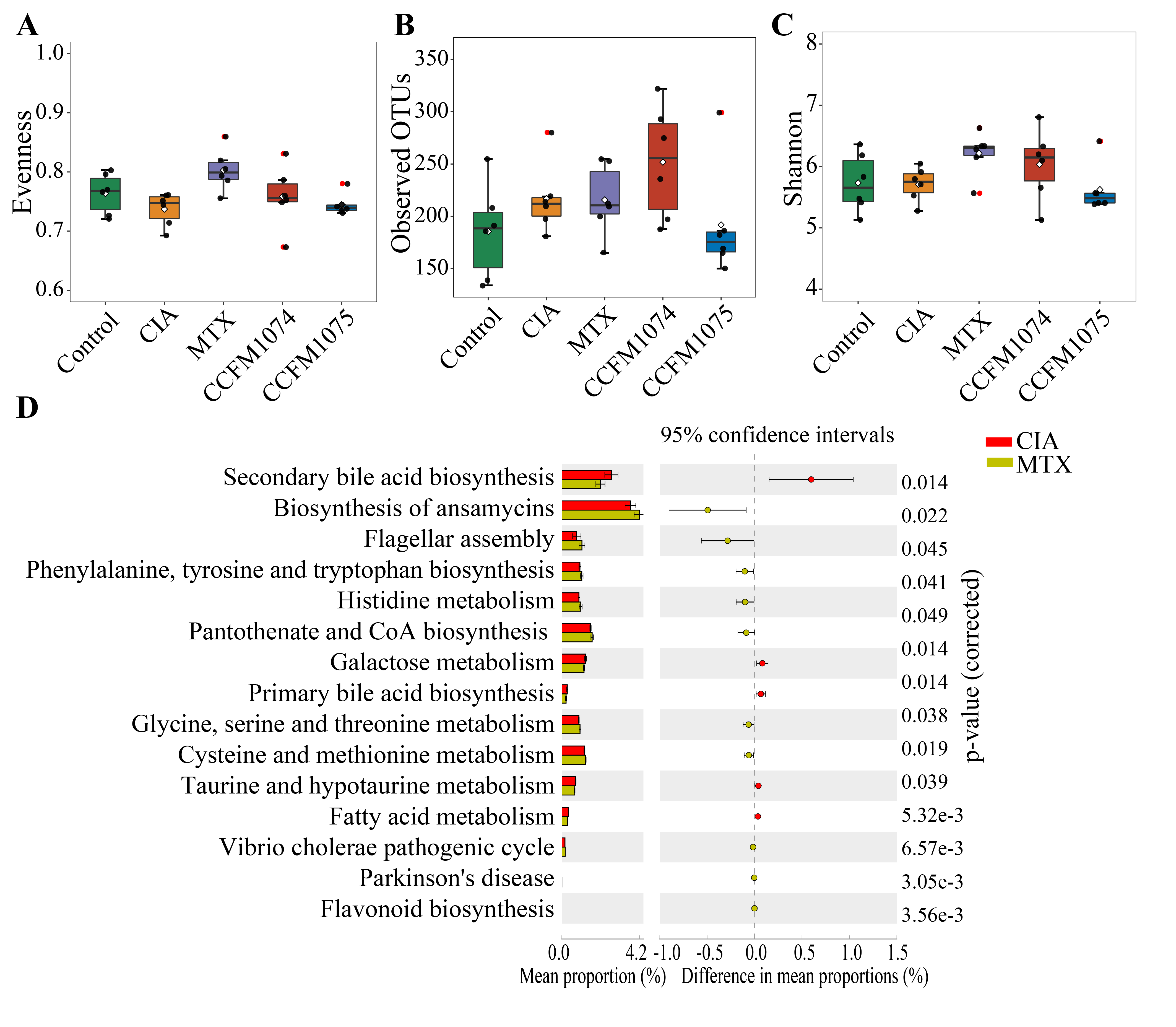

Supplement: Supplementary file 2 [file Image_2.tif]
